# Cardiorespiratory fitness decreases the odds for subclinical carotid plaques in apolipoprotein *e4* homozygotes

**DOI:** 10.1038/s41598-022-23075-2

**Published:** 2022-11-10

**Authors:** Jose Luis Perez-Lasierra, José A. Casajús, Alejandro Gonzalez-Agüero, José Miguel Arbones-Mainar, José A. Casasnovas, Martin Laclaustra, Belén Moreno-Franco

**Affiliations:** 1 Department of Physiatry and Nursing, Universidad de Zaragoza, Zaragoza, Spain; 2 GENUD (Growth, Exercise, Nutrition and Development) Research Group, Zaragoza, Spain; 3 EXERNET Red de Investigación en Ejercicio Físico Y Salud, Zaragoza, Spain; 4 Centro de Investigación Biomédica en Red de Fisiopatología de La Obesidad Y Nutrición (CIBEROBN), Madrid, Spain; 5 Instituto de Investigación Sanitaria de Aragón (IIS Aragón), Zaragoza, Spain; 6 Instituto Aragones de Ciencias de La Salud (IACS), Zaragoza, Spain; 7 Department of Medicine Psychiatry and Dermatology, Universidad de Zaragoza, Zaragoza, Spain; 8 Centro de Investigación Biomédica en Red de Enfermedades Cardiovasculares (CIBERCV), Madrid, Spain; 9 Department of Microbiology, Pediatrics, Radiology and Public Health, Universidad de Zaragoza, Zaragoza, Spain; 10 Department of Microbiology, Pediatrics, Radiology and Public Health, Universidad de Zaragoza, C/Domingo Miral S/N, 50009 Zaragoza, Spain

**Keywords:** Genetic predisposition to disease, Atherosclerosis, Lifestyle modification, Ultrasonography

## Abstract

Some studies suggest that being an apolipoprotein e4 (*APOE e4*) carrier increases the risk of atherosclerosis, and others suggest that cardiorespiratory fitness (CRF) could play a key role in atherosclerotic prevention. Our aim was to analyze the association of *APOE e4* with carotid atherosclerosis and the association of CRF with atherosclerosis in *APOE e4* carriers. A cross-sectional analysis based on a subsample of 90 participants in the Aragon Workers’ Health Study was carried out. Ultrasonography was used to assess the presence of plaques in carotid territory; the submaximal Chester Step Test was used to assess CRF; and behavioral, demographic, anthropometric, and clinical data were obtained by trained personnel during annual medical examinations. *APOE e4e4* participants were categorized into Low-CRF (VO_2max_ < 35 mL/kg/min) and High-CRF (VO_2max_ ≥ 35 mL/kg/min) groups. After adjusting for several confounders, compared with *APOE e3e3*, those participants genotyped as *APOE e3e4* and *APOE e4e4* showed an OR = 1.60 (95% CI 0.45, 5.71) and OR = 4.29 (95% CI 1.16, 15.91), respectively, for carotid atherosclerosis. Compared to Low-CRF *APOE e4e4* carriers, the odds of carotid plaque detection were 0.09 (95% CI 0.008, 0.98) times lower among High-CRF *APOE e4e4* carriers. The *APOE e4e4* genotype was associated with increased carotid atherosclerosis. However, CRF is a modifiable factor that may be targeted by *APOE e4e4* to decrease the elevation of atherosclerotic risk due to this genetic condition.

## Introduction

Atherosclerosis is a chronic arterial disease and a major cause of death. The first functional and pathological changes appear in early to mid-life and slowly progress over time^1^, conditioned by several modifiable behaviors^2,3^. However, other potential nonmodifiable risk factors could play an important role in this progression.

The apolipoprotein E epsilon 4 allele (*APOE e4*) is one of the best-investigated genetic factors related to cognitive function and cardiovascular disease (CVD)^4–6^, probably due to its association with elevated levels of low-density lipoprotein cholesterol (LDL-c) and plasma triglycerides. Although the presence of *APOE e4* has been related to increased carotid intima media thickness (cIMT)^7,8^, its association with the presence of peripheral atherosclerosis continues to be the subject of debate. In this context, while some studies have reported no associations between *APOE e4* and the presence of atheroma plaques^9–12^, others have established that *APOE e4* could be a genetic risk factor, especially in men^13–16^.

Cardiorespiratory fitness (CRF) reflects the integrated ability to transport oxygen from the atmosphere to mitochondria to perform physical work and is a strong and independent marker of risk for adverse health outcomes, including all-cause mortality^17^. Moreover, the recent literature suggests that CRF levels may be involved in the appearance and development of subclinical atherosclerosis^18,19^. Although several studies have reported inverse associations between CRF and cIMT^20,21^, and between CRF and artery calcification^22^, only a few studies have investigated the association between CRF and subclinical atherosclerosis^18,20^, and to our knowledge, no study has investigated the last association in *APOE e4* carriers.

Therefore, the aims of this study were, first, to analyze the association of *APOE e4* carrier status with carotid atherosclerosis, and second, to analyze the association of CRF with carotid atherosclerosis in *APOE e4* carriers.

## Methods

### Study participants

This cross-sectional analysis was carried out in a subsample of participants belonging to the Aragon Workers’ Health Study (AWHS), a longitudinal cohort study based on data from the annual health exams of 5678 workers of a car assembly plant^23^. These workers were mainly males (93.5%). Among the total genotyped AWHS sample (n = 5322), those individuals genotyped as *APOE e4e4* (n = 46) were invited to undergo CRF, physical activity (PA), and subclinical atherosclerosis measurements and to complete diet and behavior questionnaires. Some of the potential participants refused to participate (n = 16), so a total of 30 participants genotyped as *APOE e4e4* were assessed and randomly matched by age, sex, educational level and smoking habits with 30 AWHS participants genotyped as *APOE e3e4* and 30 AWHS participants genotyped as *APOE e3e3* who were invited to undergo the measurements and complete the questionnaires. Therefore, the final sample subjected to this analysis was composed of 90 participants.

### Carotid ultrasound

A Philips IU22 ultrasound system (Philips Healthcare, Bothell, WA, USA) was used to assess the presence of plaques in the carotid territory on both sides (right and left), which were considered together as a single site, as the two sides share similar hemodynamics. Ultrasound images were acquired with linear high-frequency 2-dimensional probes (Philips Transducer L9-3, Philips Healthcare), using the Bioimage Study protocol^24^. A plaque was defined as a focal structure that protruded into the lumen of the carotid artery at least 0.5 mm or was ≥ 50% thicker than the surrounding intima media thickness. All measurements were analyzed using electrocardiogram gated frames corresponding to end-diastole (R-wave)^25^. The presence of subclinical atherosclerosis was defined as the presence of at least 1 plaque in the carotid territory. This methodology has been used and described previously^26^.

### Physical activity

PA assessment was performed using an ActiGraph GT3X+ accelerometer (ActiGraph, Pensacola, FL, USA). Participants were required to wear the device on their nondominant wrist for 7 consecutive days. Accelerometers were initialized to record accelerations at 30 Hz with a dynamic range of ± 6 G. The acceleration records were downloaded and processed with ActiLife v.6.13.4 software (ActiGraph, Pensacola, FL, USA). Time in sedentarism (SED) and PA intensities were classified using thresholds previously proposed by Montoye^27^ for the nondominant wrist: (a) SED: ≤ 2859 counts/min, (b) light PA (LPA): 2860–3940 counts/min, (c) moderate PA (MPA): 3941–5612 counts/min, (d) vigorous PA (VPA): ≥ 5613 counts/min, or (e) moderate to vigorous PA (MVPA): ≥ 3941 counts/min. Participant records were considered valid when they covered at least 10 h/day during ≥ 4 days, requiring at least 3 weekdays and 1 weekend day.

### Cardiorespiratory fitness assessment

CRF assessment was performed through a submaximal multistep test, the Chester Step Test (CST)^28^. The CST required participants to step up and down on a single step to a metronome beat (a prerecorded audio beat). The stepping rate started at 15 steps/min for 2 min (first level) and increased 5 steps/min every 2 min until the fifth level (end of the test) or until participants reached a heart rate of 80% of the predicted maximum (220—age), whichever came first^28^. At the end of each level, the heart rate and rating of perceived exertion were recorded. Maximum oxygen uptake (VO_2max_) was predicted by plotting the recorded heart rates on a graphical datasheet, where a visual best-fit line was drawn between data points, projecting the line up to the predicted maximum heart rate and then estimating the matching oxygen uptake value. Among the 30 *e4e4* carriers, two CRF groups were created using the median as a cutoff, referred to as the Low-CRF group (VO_2max_ ≤ 35 mL/kg/min) and the High-CRF group (VO_2max_ > 35 mL/kg/min).

### APOE genotyping

DNA isolation from whole blood was performed by using the FlexiGene DNA AGF3000 kit (Qiagen, Valencia, CA, USA) on an AutoGenFlex 3000 workstation (Autogen, Holliston, MA, USA), and genotyping was carried out in the Genetics Unit-Parque Científico de Madrid (Madrid, Spain). DNA samples were spotted onto 384 plates using a Beckman BioMek 2000 automated liquid handler (Beckman High Wycombe, UK) and were diluted in a mixture consisting of TaqMan Genotyping MasterMix (Applied Biosystems, Foster City, California) and a mixture of premade TaqMan SNP genotyping assays: C_3084793_20 (rs429358) and C_904973_10 (rs7412) (Applied Biosystems). qPCR assays were performed in an HT7900 Fast Real-Time PCR System (Applied Biosystems), and SDS 2.4 software (Applied Biosystems) was used for genotype calling. Samples with known genotypes as well as negative amplification blanks were included within each run to serve as positive/negative controls and assist in genotyping. This methodology has been used and described previously^29^.

### Mediterranean diet adherence

Diet was assessed based on a 136-item semiquantitative food frequency questionnaire (FFQ) previously validated in Spain^30^. The Alternate Mediterranean Dietary Index (aMED) score was calculated based on a scale including nine components: whole grains, vegetables (excluding potatoes), fruits (juices included), legumes, nuts, fish, the ratio of monounsaturated fats to saturated fats, red and processed meats, and alcohol. The total aMED score ranges from 0 to 9, with higher scores reflecting greater Mediterranean diet adherence.

### Sociodemographic, clinical and biological data

Study participants reported their age, sex, and educational level. The clinical data included weight, height, body mass index (BMI), blood pressure, medical history, and the current use of medication. Laboratory measurements were performed on blood samples collected under fasting conditions (> 8 h). Total cholesterol, high-density lipoprotein cholesterol (HDL-c), triglycerides, and fasting serum glucose concentrations were determined via enzyme analysis using the ILAB 650 analyzer of Instrumentation Laboratory (Bedford, MA, USA). Non-HDL-c was calculated by subtracting the HDL-c value from the total cholesterol value. LDL-c was calculated using the Friedewald formula^31^ when triglyceride levels were < 400 mg/dL. We defined arterial hypertension as systolic blood pressure ≥ 140 mmHg, diastolic blood pressure ≥ 90 mmHg, or self-reported use of antihypertensive medication^32^. Dyslipidemia was defined as total cholesterol ≥ 240 mg/dL, LDL-c ≥ 160 mg/dL, HDL-c < 40 mg/dL, or self-reported use of lipid-lowering drugs^33^. Diabetes was defined as fasting serum glucose ≥ 126 mg/dL or self-reported treatment with hypoglycemic medication^32^. Smoking habits were categorized as follows: current smoker if the participant reported having smoked in the last year, former smoker if the participant had smoked at least 50 cigarettes in his lifetime but not in the last year, and never smoker. This methodology has been used and described previously^26^.

### Statistical analyses

Continuous variables are presented as the mean and standard deviation (SD), and categorical variables are presented as percentages and numerical values. The presence of atherosclerotic plaque in carotid arteries was fitted with logistic regression models depending on genotype and adjusted for BMI, hypertension, dyslipidemia, diabetes, MVPA, VO_2max_, and aMED score. Odds ratios (ORs) for the presence of carotid plaques for each genotype group were calculated using the *APOE e3e3* genotype as a reference. In secondary analysis for linear trends for the presence of *e4* alleles, *APOE* genotypes were analyzed as a continuous variable (*APOE e3e3* = 0, *APOE e3e4* = 1, *APOE e4e4* = 2). Additionally, ORs for the presence of carotid plaques in each CRF group were calculated in *APOE e4e4* carriers using the Low-CRF group as a reference. A two-sided p value lower than 0.05 was considered statistically significant. Before conducting the analyses and after considering the missing at random or missing completely at random nature, participants with missing data in VO_2max_ (n = 13) or MVPA (n = 8) (Supplementary Table 1) were imputed using the expectation maximization algorithm^34^. The variables used to impute missing data were the genotype, age, sex, educational level, smoking status, BMI, VO_2max_, and a compendium of accelerometry variables related to PA and sedentary behavior. Statistical analysis was performed using SPSS statistical software ver. 24.0 (IBM Corp, Armonk, NY, USA).

### Ethical approval

All methods were carried out in accordance with relevant guidelines and regulations. The study was approved by the Clinical Research Ethics Committee of Aragon (CEICA) (PI07/09), and all participants provided written informed consent.

## Results

The sample included 90 men (mean age 60.0; SD 4.5 years). The 30 *APOE e4e4* participants showed higher systolic blood pressure than the *APOE e3e3* participants as well as greater adherence to the Mediterranean diet than *APOE e3e4* participants. Baseline characteristics of study participants according to *APOE* genotype are shown in Table 1. Additionally, baseline characteristics of *APOE e4e4* participants according to the presence of carotid plaques are shown in Supplementary Table 2.

**Table 1:** Baseline characteristics of study participants according to *apolipoprotein E genotype*.

	Overall	*APOE e3e3*	*APOE e3e4*	*APOE e4e4*	p value
N	90	30	30	30	
Age, years	60.0 (4.5)	60.4 (3.6)	60.0 (3.8)	59.7 (5.8)	0.842
BMI, kg/m^2^	27.4 (3.0)	27.4 (2.9)	27.6 (3.5)	27.3 (5.8)	0.934
Total cholesterol, mg/dL	219.6 (40.4)	214.6 (43.1)	220.5 (43.9)	223.7 (34.6)	0.690
HDL-c, mg/dL	50.4 (10.1)	51.7 (10.3)	49.8 (11.4)	49.6 (8.7)	0.694
Non-HDL-c, mg/dL	169.7 (37.1)	162.9 (39.2)	170.1 (39.7)	176.1 (32.3)	0.405
LDL-c, mg/dL	142.2 (34.3)	138.2 (34.8)	141.4 (34.8)	147.6 (34.0)	0.596
Triglycerides, mg/dL	140.8 (79.5)	124.2 (53.6)	145.6 (62.4)	152.7 (110.4)	0.369
Glucose, mg/dL	104.4 (22.6)	100.3 (21.0)	102.7 (14.4)	110.1 (29.4)	0.229
SBP, mmHg	137.1 (16.8)	133.5 (14.6)	133.8 (16.2)	143.9 (18.0)*	**0.024**
DBP, mmHg	85.3 (8.9)	84.4 (7.9)	84.7 (8.4)	87.0 (10.3)	0.459
VO_2__max_, mL/kg/min	38.8 (7.9)	38.9 (7.8)	40.7 (8.2)	36.8 (7.7)	0.172
MVPA time, min/day	109.6 (81.2)	103.3 (69.3)	112.0 (87.4)	113.5 (87.9)	0.872
Hypertension, %	67.8 [61]	63.3 [19]	70.0 [21]	70.0 [21]	0.816
Dyslipidemia, %	70.0 [63]	63.3 [19]	66.7 [20]	80.0 [24]	0.329
Diabetes, %	12.2 [11]	13.3 [4]	6.7 [2]	16.7 [5]	0.484
Carotid plaque, %	53.3 [48]	36.7 [11]	60.0 [18]	63.3 [19]	0.079
aMED score, points	4.12 (1.8)	4.07 (1.5)	3.50 (2.1)	4.80 (1.6)**	**0.019**

Significant p values are in bold.

*BMI* body mass index, *HDL-c* high-density lipoprotein cholesterol, *LDL-c* low-density lipoprotein cholesterol, *SBP* systolic blood pressure, *DBP* diastolic blood pressure, *MVPA* moderate to vigorous physical activity, *aMED score* alternate Mediterranean dietary index. Values are the mean (SD) or % [number].

*Indicates a significant difference (p < 0.05) from the *APOE e3e3* group.

**Indicates a significant difference (p < 0.05) from the *APOE e3e4* group.

### Odds ratios for carotid atherosclerosis by genotype

At least one carotid plaque was found in 48 participants (53.3% of the overall sample), including 36.7% of the *APOE e3e3* group, 60.0% of the *APOE e3e4* group, and 63.3% of the *APOE e4e4* group (Table 1). The odds of showing a carotid plaque were 4.29 times higher (95% CI 1.16, 15.91, p < 0.05) among those participants genotyped as *APOE e4e4* than those genotyped as *APOE e3e3* after adjusting for BMI, hypertension, dyslipidemia, diabetes, MVPA, VO_2max_, and aMED score (Table 2).

**Table 2:** Odds ratio (95% CI) for the presence of plaque in carotid territory by *apolipoprotein E* genotype.

	Genotype		
	*APOE e3e3*	*APOE e3e4*	*APOE e4e4*
Number with carotid plaques/Total	11/30	18/30	19/30
Unadjusted	1.00 (ref)	2.59 (0.91, 7.34)	2.98 (1.04, 8.53)
Model 1	1.00 (ref)	3.05 (1.01, 9.28)	3.38 (1.08, 10.53)
Model 2	1.00 (ref)	3.08 (1.01, 9.34)	3.42 (1.09, 10.67)
Model 3	1.00 (ref)	3.11 (1.02, 9.49)	3.30 (1.05, 10.34)
Model 4	1.00 (ref)	3.12 (1.02, 9.53)	3.34 (1.06, 10.49)
Model 5	1.00 (ref)	1.60 (0.45, 5.71)	4.29 (1.16, 15.91)

Model 1: Adjusted for body mass index, hypertension, dyslipidemia, and diabetes.

Model 2: Model 1 additionally adjusted for MVPA (min/day).

Model 3: Model 1 additionally adjusted for VO_2__max_ (mL/kg/min).

Model 4: Model 1 additionally adjusted for MVPA (min/day) and VO_2__max_ (mL/kg/min).

Model 5: Model 4 additionally adjusted for aMED score (0–9).

Although the odds of having a carotid plaque also tended to be higher in the *APOE e3e4* participants than in the *APOE e3e3* participants (adjusted ORs 1.60; 95% CI 0.45, 5.71, p = 0.46), the difference was not statistically significant (Table 2). However, a secondary analysis showed a linear trend with increasing odds of carotid plaque per unit change in genotype among *APOE e3e4* and *APOE e4e4* participants in relation with those *APOE e3e3* participants, (ORs 1.92; 95% CI 1.07, 3.44, p = 0.03) after adjusting the results for BMI, hypertension, dyslipidemia, diabetes, MVPA, VO_2max_, and aMED score.

### Odds ratios for carotid atherosclerosis according to CRF status in APOE e4e4 participants

Among the 15 *APOE e4e4* participants who were in the Low-CRF group, 12 presented at least one carotid plaque, and among the 15 *APOE e4e4* participants who were in the High-CRF group, at least one carotid plaque was present in 7 participants (Table 3). The odds of having a carotid plaque were 0.09 times lower (95% CI 0.008, 0.98, p < 0.05) among those *APOE e4e4* participants who presented High-CRF (VO_2max_ > 35 mL/kg/min) than those *APOE e4e4* participants allocated to the Low-CRF group (VO_2max_ ≤ 35 mL/kg/min) after adjusting for age, BMI, hypertension, dyslipidemia, diabetes, smoking status, MVPA, and aMED score (Table 3).

**Table 3:** Odds ratio (95% CI) for the presence of plaque in carotid territory according to cardiorespiratory fitness group in *apolipoprotein e4e4* carriers (n = 30).

	Cardiorespiratory fitness (CRF)	
	Low-CRF	High-CRF
Number with carotid plaques/Total	12/15	7/15
Unadjusted	1.00 (ref)	0.22 (0.04, 1.11)
Model 1	1.00 (ref)	0.13 (0.16, 1.10)
Model 2	1.00 (ref)	0.09 (0.008, 0.98)

Low-CRF: VO_2__max_ ≤ 35 mL/kg/min; High-CRF: VO_2__max_ > 35 mL/kg/min.

Model 1: Adjusted for age, body mass index, hypertension, dyslipidemia, diabetes and smoking status (current smoker, never smoker, and former smoker).

Model 2: Additionally adjusted for MVPA (min/day) and aMED score (0–9).

To test the robustness of the results related to CRF as a potential protective factor against carotid plaques in *APOE e4e4* carriers, a sensitivity analysis was conducted with the *APOE e4e4* participants lacking imputed VO_2max_ data (n = 26) (Table 4). After creating new CRF groups using the median VO_2max_ of these 26 participants as a cutoff (median VO_2max_ = 34.3 mL/kg/min), the ORs for carotid plaques were studied. The odds of having a carotid plaque were 0.003 times lower (95% CI: 0.0001, 0.89, p < 0.05) among those *APOE e4e4* participants who presented High-CRF (VO_2max_ > 34.3 mL/kg/min) than those *APOE e4e4* participants who presented Low-CRF (VO_2max_ ≤ 34.3 mL/kg/min) after adjusting for age, BMI, hypertension, dyslipidemia, diabetes, smoking status, MVPA, and aMED score (Table 4).

**Table 4:** Odds ratio (95% CI) for the presence of plaque in carotid territory by cardiorespiratory fitness group in *apolipoprotein e4e4* carriers with no imputed VO_2__max_ data (n = 26).

	Cardiorespiratory fitness (CRF)	
	Low-CRF	High-CRF
Number with carotid plaques/Total	10/13	7/13
Unadjusted	1.00 (ref)	0.35 (0.07, 1.90)
Model 1	1.00 (ref)	0.16 (0.01, 2.61)
Model 2	1.00 (ref)	0.003 (0.0001, 0.89)

Low-CRF: VO_2__max_ ≤ 34.3 mL/kg/min; High-CRF: VO_2__max_ > 34.3 mL/kg/min.

Model 1: Adjusted for age, body mass index, hypertension, dyslipidemia, diabetes, and smoking status (current smoker, never smoker, and former smoker).

Model 2: Additionally adjusted for MVPA (min/day) and aMED score (0–9).

## Discussion

This cross-sectional analysis of middle-aged men shows that *APOE e4* homozygosity is associated with higher odds of the presence of carotid plaques than *APOE e3* homozygosity independent of other CVD and lifestyle risk factors. In addition, in *APOE e4* homozygotes, a higher CRF was associated with lower odds for carotid atherosclerosis plaques compared with a lower CRF.

Our results regarding the association between the *APOE* genotype and carotid plaques are in accordance with previous studies^13,15,16^, but only two of these studies reported results for *APOE e4e4* and *APOE e3e4* carriers separately, rather than for overall *APOE e4* carriers (*APOE e4e4* and *APOE e3e4* together)^13,16^ (included in Supplementary Table 3). The results of our study are similar to those observed by Debette et al., which proves that *APOE e4* homozygotes present higher odds of carotid plaque formation than *APOE e3* homozygotes (adjusted ORs 2.12; 95% CI 1.27, 3.53, p = 0.004) and that although the odds of carotid plaque formation tend to be higher in *APOE e3e4* carriers than in *APOE e3* homozygotes, the association is not statistically significant (ORs 1.08; 95% CI 0.93, 1.25, p = 0.33)^13^. Also, the results of our study are similar to those observed by Beilby et al., which reported a linear trend with an increasing risk of carotid plaque per unit change in genotype among men with *APOE e3e4* and *APOE e4e4* in relation to those with *APOE e3e3* men (ORs 1.72; 95% CI 1.05, 2.80, p = 0.03)^16^.

On the other hand, as mentioned above, other studies have reported no associations between *APOE e4* and atherosclerotic carotid plaques after adjusting for major confounders^9–12^. As supported by previous studies, adjusting the results based on important confounders such as smoking or hypertension seems to be crucial^35^. However, most of these studies have not adjusted their results based on relevant and usually overlooked factors, such as PA, CRF, or adherence to specific dietary patterns. This omission could be relevant to the reproducibility of the results^19,36^. Additionally, although the total samples included in these studies seem to be reasonable, the number of participants genotyped as *APOE e4e4* is very low. For example, only 10 participants among the total sample (n = 544) of the Djoussé et al. study were *APOE e4e4* carriers^12^. Likewise, most of these studies are mainly limited to presenting data separately for *APOE e3e4* and *APOE e4e4* carriers^9,10,12^, and as supported by this study and the study conducted by Debette et al. only the *APOE e4e4* polymorphism is associated with higher odds of carotid plaque formation^13^.

The exposure of *APOE e4e4* carriers to contemporary environmental conditions, such as a Western diet, sedentarism, and longer lifespans, could have led this allele to become a susceptibility allele for peripheral atherosclerosis, coronary artery disease, and several neurodegenerative diseases^37^. However, some lifestyle factors, such as PA and diet, could modulate genetic expression and seem to be key factors modulating susceptibility and reducing the elevated risks of disease and mortality associated with the *APOE e4e4* genotype^38–41^.

The health benefits of high CRF in the general population are well described in the literature^17,42^. However, the role of CRF in atherosclerosis development is underexamined. Only two studies have focused on the association of CRF with carotid plaques, revealing an inverse association^18,20^. Although higher CRF seems to be a protective factor against atherosclerosis in the general population^18,20^, no studies have investigated this potential effect in subjects with increased genetic risk of atherosclerosis, such as *APOE e4e4* carriers. To the best of our knowledge, this is the first study that has investigated the association of CRF and carotid plaques specifically in *APOE e4e4* carriers, showing that higher CRF is associated with decreased odds of atherosclerosis development. Bearing in mind that atherosclerosis represents an intermediate step toward CVD and death, this finding has high clinical and public health significance.

This study involved a modest sample size but benefited from the inclusion of a significant number of *APOE e4e4* carriers and other matched participants by randomization. In addition, the study presents the strength of high-quality data collection methods for obtaining information on subclinical atherosclerosis and other variables. However, several limitations of our study should be acknowledged. First, the cross-sectional design does not allow us to establish causal temporal links. Second, the sample includes only Southern European men, and therefore, the results may not be generalizable to women and individuals of other races or ethnic groups in the population. Third, CRF assessment was performed using a previously validated but indirect technique; therefore, VO_2max_ was estimated and was not directly measured. Fourth, cognitive status, which could modify the associations because *APOE e4e4* carriers are more vulnerable to cognitive decline, was not evaluated.

In conclusion, the *APOE e4e4* genotype but not the *APOE e3e4* genotype was associated with higher carotid atherosclerosis prevalence in men independent of traditional and other atherosclerotic risk factors, supporting the previous observations revealing this association. On the other hand, CRF is a modifiable physiological attribute that may be targeted in *APOE e4e4* carriers to decrease their increased atherosclerosis risk due to their genetic condition.

## Supplementary Information

Supplementary Tables.

## Data Availability

The data presented in this study are available on request from the corresponding author. The data are not publicly available for ethical reasons.
