# Supplementary material for: Cardiorespiratory fitness decreases the odds for subclinical carotid plaques in apolipoprotein e4 homozygotes
Source: Sci Rep. 2022 Nov 10;12:19196. doi: 10.1038/s41598-022-23075-2 (PMC9649711; doi:10.1038/s41598-022-23075-2)
Supplement: Supplementary file 1 — Supplementary Tables. [file 41598_2022_23075_MOESM1_ESM.docx]

| **Imputed variables** | **Overall** | ***APOE e3e3*** | ***APOE e3e4*** | ***APOE e4e4*** |
| --- | --- | --- | --- | --- |
| **VO_2_max,** % | 14.4 [13] | 10.0 [3] | 20.0 [6] | 13.3 [4] |
| **MVPA,** % | 8.9 [8] | 10.0 [3] | 6.7 [2] | 10.0 [3] |

**Supplementary Table 1** Number of participants with missing and imputed data by variable and by genotype. Values are % [number].

|  | **Overall** | ***With plaque*** | ***Without plaque*** | **p value** |  |
| --- | --- | --- | --- | --- | --- |
| **N** | 30 | 19 | 11 |  | |
| **Age,** years | 59.7 (5.8) | 60.8 (3.2) | 57.8 (8.5) | 0.183 | |
| **BMI**, kg/m^2^ | 27.3 (5.8) | 27.3 (2.5) | 27.2 (3.3) | 0.875 | |
| **Total cholesterol,** mg/dL | 223.7 (34.6) | 224.7 (39.6) | 222.2 (26.0) | 0.855 | |
| **HDL-c,** mg/dL | 49.6 (8.7) | 48.2 (8.5) | 52.0 (9.0) | 0.257 | |
| **Non-HDL-c,** mg/dL | 176.1 (32.3) | 176.5 (36.0) | 175.5 (26.7) | 0.940 | |
| **LDL-c,** mg/dL | 147.6 (34.0) | 150.7 (35.5) | 141.7 (32.0) | 0.529 | |
| **Triglycerides,** mg/dL | 152.7 (110.4) | 138.1 (83.9) | 176.6 (145.4) | 0.371 | |
| **Glucose,** mg/dL | 110.1 (29.4) | 119.4 (34.2) | 95.0 (5.5) | **0.027** | |
| **SBP,** mmHg | 143.9 (18.0) | 148.0 (16.7) | 136.7 (18.6) | 0.099 | |
| **DBP,** mmHg | 87.0 (10.3) | 86.5 (8.1) | 87.9 (13.6) | 0.719 | |
| **VO_2_max,** mL/kg/min | 36.8 (7.7) | 34.2 (5.3) | 41.3 (9.3) | **0.012** | |
| **MVPA time,** min/day | 113.5 (87.9) | 99.4 (80.6) | 138.1 (98.4) | 0.252 | |
| **Hypertension,** % | 70.0 [21] | 73.7 [14] | 63.6 [7] | 0.563 | |
| **Dyslipidemia,** % | 80.0 [24] | 78.9 [15] | 81.8 [9] | 0.850 | |
| **Diabetes,** % | 16.7 [5] | 26.3 [5] | 0.0 [0] | 0.062 | |
| **Current smokers,** % | 40.0 [12] | 31.6 [6] | 54.5 [6] | 0.438 | |
| **Former smokers,** % | 40.0 [12] | 47.4 [9] | 27.3 [3] | 0.438 | |
| **Never smokers,** % | 20.0 [6] | 21.1 [4] | 18.2 [2] | 0.438 | |
| **aMED score,** points | 4.80 (1.6) | 4.89 (1.6) | 4.64 (1.7) | 0.679 | |

**Supplementary Table 2** Baseline characteristics of *APOE e4e4* participants according to the presence of carotid plaque. BMI: body mass index; HDL-c: high-density lipoprotein cholesterol; LDL-c: low-density lipoprotein cholesterol; SBP: systolic blood pressure; DBP: diastolic blood pressure; MVPA: moderate to vigorous physical activity; aMED score: alternate Mediterranean dietary index. Values are mean (SD) or % [number].

|  | **Genotype** | |
| --- | --- | --- |
|  | ***APOE e3e3*** | ***APOE e3e4 & e4e4*** |
| **Number with carotid plaques/Total** | 11/30 | 37/60 |
| Unadjusted | 1.00 (ref) | 2.78 (1.12, 6.88) |
| Model 1 | 1.00 (ref) | 3.21 (1.21, 8.52) |
| Model 2 | 1.00 (ref) | 3.23 (1.21, 8.61) |
| Model 3 | 1.00 (ref) | 3.20 (1.20, 8.52) |
| Model 4 | 1.00 (ref) | 3.22 (1.21, 8.59) |
| Model 5 | 1.00 (ref) | 3.25 (1.22, 8.69) |

**Supplementary Table 3** Odds ratio (95% CI) for the presence of plaque in carotid territory by *apolipoprotein E* genotype.

Model 1: Adjusted for body mass index, hypertension, dyslipidemia, and diabetes.

Model 2: Model 1 additionally adjusted for MVPA (min/day).

Model 3: Model 1 additionally adjusted for VO_2_max (mL/kg/min).

Model 4: Model 1 additionally adjusted for MVPA (min/day) and VO_2_max (mL/kg/min).

Model 5: Model 4 additionally adjusted for aMED score (0-9)
